# Characterization of Composite Freeze-Dried Aerogels with Simulant Lunar Regolith for Space Applications

**DOI:** 10.3390/ma16175797

**Published:** 2023-08-24

**Authors:** Laura Borella, Andrea Rozo, Claire Perfetti, Carlo Saverio Iorio

**Affiliations:** Centre for Research and Engineering in Space Technologies, Université Libre de Bruxelles, 1050 Brussels, Belgium; andrea.rozo@ulb.be (A.R.); claire.perfetti@ulb.be (C.P.); carlo.iorio@ulb.be (C.S.I.)

**Keywords:** Moon, aerogels, lunar night, space insulators, ISRU, lunar regolith

## Abstract

Recently, the goal of space exploration has shifted from the incognito of the solar system to the Moon. Concepts like human permanence on the Moon and thermal protective structures made with ISRU (in situ resource utilization) of raw materials have started to be implemented. By limiting the need to launch supplies from the Earth, the paradigm of spaceflight is changed, privileging the vanguard of the utilisation of resources in situ. Still, the main challenges of surviving the radiation dose and the cryogenic temperatures of the lunar night remain. Recent studies have demonstrated how innovative composite materials can help reduce the temperature stress on exploration vehicles. This research presents the material properties of aerogel insulating materials combined with LHS (lunar highlands simulant) regolith obtained by freeze frying. Organic-based aerogels with different percentages of LHS have been analysed in terms of material, morphology, and thermal properties.

## 1. Introduction

The implementation of insulator materials to survive in space’s harsh environment is still an open challenge; additionally, their production in a non-terrestrial environment is yet unknown. This represents the main obstacle to human settlement on extraterrestrial bodies. After several missions, from Apollo to the most recent Perseverance, the Moon and Mars are the next two targets of space exploration. Knowledge gathered during the last 70 years gave a detailed understanding of soil composition, environmental appearance, light cycles, and climate conditions, among which repeated hazardous events. When humans return to the Moon, in situ resource utilization (ISRU) of the lunar regolith will allow a more efficient, less costly, and more sustainable human presence to be achieved. Maintaining a human presence on the Moon will require methods to mitigate lunar dust, provide protection from micrometeoroid impact, and reduce astronaut exposure to radiation [1]. Among the most effective insulators for space are multi-layer insulators (MLI), which consist of several layers of thin sheets and are commonly used in spacecraft and cryogenic applications [2]. Aerogel materials, with their very low density and high thermal resistance, have been used in various space missions due to their exceptional insulating properties [3]. Cork also has thermal insulation properties, such as low thermal conductivity, making it effective against temperature extremes [4]. Its light weight is critical for weight-critical space missions, and its flexibility and compressibility allow it to conform to irregular shapes or gaps, which is beneficial for insulating complex structures [4]. In addition, cork generally has low outgassing, which is essential to preventing contamination of sensitive equipment. However, there are considerations and challenges associated with using cork in space. Its radiation resistance may not be as robust as other specialised space insulators such as aerogels or silica fibre-reinforced Composites (SiFRC), which combine silica fibres with a polymer matrix [5]. In addition, the long-term performance of cork in the harsh space environment, including exposure to ultraviolet radiation, vacuum, and micrometeoroid impacts, is not well understood. The sustainability and resource availability of cork oak trees on other celestial bodies would be critical for their use in non-terrestrial environments. Resin, another potential space insulation material, offers a wide range of applications and properties relevant to space insulators. Certain resins have low thermal conductivity, making them suitable for thermal insulation in space applications [6]. They can also exhibit high strength and durability, which are essential to withstand the rigours of the space environment [7]. In addition, resins are relatively light, favourable for reducing spacecraft mass, and can be easily moulded into various shapes and forms, enabling customization and fitting into complex structures.

In situ resource recovery could produce resins for space if precursors are available on celestial bodies. Various strategies for insulation in non-terrestrial environments have been proposed. Given the quantity of loose surface debris on the Moon and Mars, the use of regolith is a promising technique [8]. Researchers have investigated using regolith to make insulating materials by processing it to extract silica, which may then be used to make silica fibre-reinforced composites (SiFRC) [9] or other composites. Moreover, the production of aerogels, another effective insulator, could be achieved using in situ resources on the Moon, Mars, or asteroids. Studies have investigated the possibility of using aerogels from materials available in the Martian atmosphere, such as carbon dioxide [10], and silica extracted from regolith [11]. Furthermore, the application of in situ 3D printing techniques offers a promising way to manufacture insulating materials using locally available resources. Specifically, 3D printers equipped with regolith processing capabilities could produce structures [12] made of regolith-based materials, providing an efficient and customizable insulation solution for space missions [13]. Considering the challenges and requirements of space environments, the integration of these production methods and insulating materials should be carefully studied and tailored to meet the specific needs of each space mission. Careful consideration of the specific properties and challenges of each insulation material is essential in designing effective and durable insulation systems for space exploration. However, the time of exposure to cryogenic temperatures is also consistent since one lunar night lasts for almost 14.5 Earth days, and so does a Moon day. The challenges associated with materials selection for space applications are closely linked to the formidable conditions of space, characterised by extreme temperatures, radiation exposure, and the potential for micrometeoroid impacts. Several dust mitigation technologies have been investigated in the literature as solutions in the lunar environment. These include coatings, electrostatic discharge films, work function matching, brushing, blowing, electrodynamic methods, vibration, ultrasonic techniques, and zapping with an electron beam [14]. Passive technology solutions can include HEPA (high-efficiency particulate air) filters, cyclone separators, soft walls, low-energy surface coatings, coveralls, dust tarpaulins, brushes, tape, and wipes. Active technology solutions can include electrostatics, compressed air, high-energy surface coatings, and vacuum cleaners [15]. Among the many complexities encountered in space missions, achieving effective thermal control for electronic components, particularly batteries, emerges as a prominent concern. Despite the introduction of multi-layer insulators (MLIs) with robust insulation properties to protect batteries [16], maintaining optimal operating conditions in nighttime temperature scenarios remains an unresolved dilemma. This challenge is especially critical for exploration vehicles lacking nuclear active thermal control systems such as RTGs (radioisotope thermal generators), as there is currently no exact solution to ensure temperatures within the designated lower operating range of −40 °C [17]. Deviation from the prescribed operating ranges can result in system failure or necessitate the premature termination of a mission.

Additionally, these materials must possess the mechanical strength to withstand conditions related to launch vibrations and potential impacts from space debris while maintaining their integrity and properties. Of all materials used for space applications, aerogels are a class of porous materials with a remarkable combination of properties, including ultra-low density, high surface area, and excellent thermal insulation according to the review by Hu, L., He, R., Lei, H. et al. [18].

Aerogels can be broadly classified into two categories: inorganic and organic aerogels [19]. Inorganic aerogels are typically made from metal oxides such as silica, alumina, or titania. These materials are widely used in applications such as catalysis, sensors, and energy storage, as well as in space exploration, due to their ability to withstand extreme conditions. On the other hand, organic aerogels are derived from organic polymers, such as cellulose or melamine-formaldehyde, and are known for their low density, high mechanical strength, and tunable properties. In industries including aircraft, insulation, and biomedical engineering, where their low weight and mechanical attributes make them desirable for structural and functional applications, organic aerogels have found use. A combination of the two is also widely implemented and goes by the name of hybrid aerogels [19]. All varieties of aerogel are produced using the same method, although the technique varies depending on the materials used. The polymerization process and the sol-gel preparation are two different ways to make aerogels. In the first, a sol or colloidal suspension is created, which is subsequently gelled to create an aerogel [20]. Sol–gel chemistry is generally the preparation of inorganic polymers or ceramics from solution via a transformation from liquid precursors to a sol and finally to a network structure called a ‘gel’ [21]. The sol is typically made by dissolving metal alkoxides or other precursors in a solvent, which is then hydrolyzed and condensed to form a network of interconnected nanoparticles [22]. The resulting gel is then subjected to supercritical drying to remove the solvent and produce the aerogel [23]. Contrarily, in polymerization, a network of polymers is created from monomers or oligomers and then cross-linked to create a gel. Different chemical or physical methods, such as thermal, photochemical, or radiation-induced polymerization, can be used to create cross-links [24]. Once the gel is formed, it can be dried using a supercritical drying or freeze-drying process to produce the aerogel. The advantage of organic-based aerogels lies in the cost of production and the manufacturing process, which make organic aerogels cheaper than inorganic ones [25].

Aerogels have proven useful in space missions dedicated to the gathering of extraterrestrial particles such as interstellar dust and cometary debris, as demonstrated by the Stardust mission [26]. Aerogels have been employed in a variety of cryogenic applications, particularly in specific portions of LNG (liquefied natural gas) services, by leveraging their large surface area to reduce weight and volume. These services, which include liquefaction (export) and regasification (import) terminals, operate primarily at −165 °C and are the largest cryogenic facilities [27]. NASA turned to aerogel to keep rocket fuel at cryogenic temperatures, working with industry to develop the world’s first practical, flexible aerogel blankets in the 1990s [28]. The utilization of aerogel-based materials in cryogenic thermal insulation systems is often economically justified [29]. On the latest Mars mission, the Perseverance rover carried on board the aerogel superlight insulation, minimising the power needed to keep it at operating temperatures [30]. Aerogels are becoming increasingly crucial in space systems for optimising thermal control, particularly in the harsh lunar environment. Because of their low density, high porosity, high surface area, low refractive index, low dielectric constant, outstanding thermal insulation qualities, and temperature tolerance [31], this study shows their possible utility in future lunar constructions. Aerogels allow space systems to better survive cryogenic temperatures during lunar nights, boosting thermal performance and component durability and so contributing to the successful realisation of long-lasting and dependable structures on the Moon. Hybrid aerogels with ISRU are a compromise between organic and inorganic ones, promoting sustainability while ensuring reliable and durable space systems.

## 2. Experiment Section

### Materials and Methods

For this work, it is reported the production of organic-based aerogels to lead sustainability in space, with the addition of simulant lunar regolith from the highlands produced via chemical polymerization and using a freeze dryer to ensure the sublimation of the water content. The materials used were obtained from different companies. Gelatin derived from bovine collagen via thermal, sodium alginate powder with pH at 25 °C between 5 and 8, viscosity 5.0–40.0 cps, and calcium chloride (${\mathrm{CaCl}}_{2}$) (with a molecular weight of 110.98) were procured from Sigma-Aldrich. Additionally, the lunar highlands simulant (LHS) regolith containing ultra-fine particles with a diameter of 0.053 mm was acquired from Off Planet Research (Table 1). Complementary, also the XRD compositional analysis of the purchased simulant was provided and reported below (Table 2). All chemicals and LHS were used as received. The selection of the particle size of the latter depends on the fact that larger particles, often with sharper angles, may create microfractures during the processing of the final product. By using ultra-fine particles, the risk of microfractures is minimised, and the resulting product appears more uniform. The simulant purchased is named OPRH2N [32], and the following information has been released.

The melting temperatures of the constituent materials, anorthosite and JSC-1A basalt, are approximately 1500–1600 °C [34] and 1100–1125 °C [35], respectively. The material of interest, OPRH2N, is a composite of 70% anorthosite and 30% JSC-1A basalt. However, specific data on the combined melting temperature of OPRH2N is currently lacking. It is worth noting that various factors, like heating duration, grain size distribution, and the presence of water content (if not adequately dried), can all influence these temperatures.

The procedure starts with the production of a 4% sodium alginate (SA) water solution. Sodium alginate is a hydrophilic polymer that readily dissolves in water to form a viscous solution with properties such as high elasticity, adhesiveness, and gelling ability. SA matrixes not only improve composite adsorption capacity and structural integrity, but they also reduce the loss of powdered or liquid-based adsorbents [36,37]. The development of SA composites would be a potential method for minimising our dependency on non-renewable resources while also reducing their environmental impact [36]. In the solution, the polymer chains are well dispersed in the water, forming a homogeneous solution that appears as a slightly viscous liquid. To prepare the sodium alginate solution (SA), 4% sodium alginate powder was added to deionized (DI) water at 30 °C while continuously stirring. The mixture was left on a magnetic stirrer for at least 2 h until the powder dissolved and a homogeneous solution was obtained. The solution was then degassed for 1 h to eliminate any air bubbles present [38].

Subsequently, to improve the mechanical stability of biopolymers, a common strategy is to blend two or more polymers to construct double or multiple networks [39]. About 0.4% of gelatine was added to the SA solution [40] and stirred for 2 h at a temperature of 40 °C. Alginate structure consists of linear copolymers having blocks of D-mannuronic acid (M) and L-guluronic acid (G) residues [41,42]. The blocks are composed of consecutive G residues (GGGGGG), consecutive M residues (MMMMMM), and alternating M and G residues (GMGMGM). When in contact with different divalent cations, such as calcium (${\mathrm{Ca}}^{2+}$), barium (${\mathrm{Ba}}^{2+}$), and magnesium (${\mathrm{Mg}}^{2+}),$ the G blocks on different polymer chains form ionic crosslinks via –COO– [41,43], which results in the formation of the hydrogel matrix. A cross-linked network is formed by electrostatic interaction and hydrogen bonding due to the good compatibility of SA and gelatine. This solution served as the organic base, to which LHS was added at varying percentages of 20%, 40%, and 60%. LHS was stirred for approximately 5 min until it reached visual homogeneity. However, as the concentration of the LHS approached or exceeded 60%, the solution became excessively powdery and brittle, losing its mechanical and density properties. Because the interior structure of SA/gelatine aerogels is unable to entirely accept excess powder of LHS, it disperses in the calcium-water solution. When the percentage of LHS exceeds 60, the creation of porous structures becomes more difficult, owing to LHS occupying spaces that would normally be filled by frozen water crystal sites. Excess LHS interferes with the natural formation process of porous structures, affecting the overall composition and characteristics of the material. At higher concentrations of LHS, the aerogel does not form properly because there is an excess of the dusty component dispersed in the polymerization solution. Porous formation does not take place because the LHS occupies what are supposed to be frozen water crystal sites. To create the desired shapes, the solution was poured into custom-designed and 3D printed in-house PLA moulds. The mould size was chosen considering the limitations of the container for the freeze dryer as well as the fact that they were placed parallel to the bottom of the container to ensure uniformity during sublimation. The dimensions of the samples are around 5 cm long and 6 mm thick. Once the solution was poured into the moulds, they were frozen in liquid nitrogen vapours to avoid sedimentation of the simulant regolith grains. The effect of LN is to instantly freeze the heavier LHS particles when mixed with the lighter SA/gelatine solution prior to polymerization. This process prevents sedimentation of the LHS particles under the influence of gravity during the polymerization time. The instantaneous freezing of the particles maintains a uniform distribution, preventing the heavier particles from settling to the bottom. This technique helps to produce a homogeneous hybrid aerogel. The physical cross-linking [44] was achieved by exposing the solution in the moulds to a 2.5% calcium chloride (${\mathrm{CaCl}}_{2}$) solution [38] in DI water. ${\mathrm{CaCl}}_{2}$ is in charge of initiating the polymerization reaction in the mixture. If ${\mathrm{CaCl}}_{2}$ was not present, the mixture would not gel and would stay liquid. As a result of the crosslinking of polymer chains, a hydrogel is formed. Its presence is required for the liquid combination to be converted into a solid hydrogel structure. During this process, a homogeneous network is formed, which influences the increase in strength of the aerogel [39]. The ${\mathrm{Ca}}^{2+}$ ions from the ${\mathrm{CaCl}}_{2}$ solution penetrated the core of the material over a period of 5 h. The advantage of starting with a hydrogel is that it allows one to adapt and produce the material in different shapes and dimensions.

**Figure 1:**
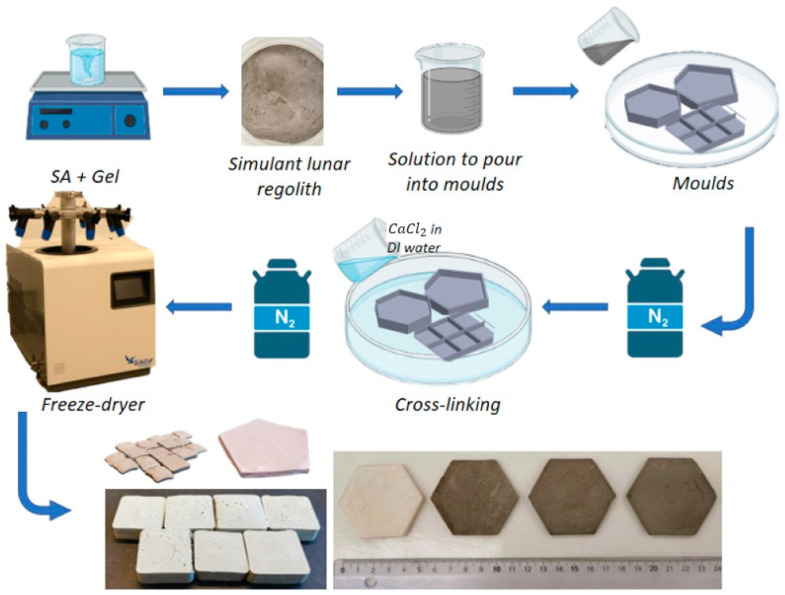
Schematic diagram of the preparation process of organic-based aerogels with simulant lunar regolith.

Figure 1 schematically shows the development process of freeze-dried aerogels with LHS.

Thanks to the initial liquid state of the mixture, it can be easily poured into various sizes and types of moulds, providing flexibility in shaping the material according to different requirements. The mixture turns into a gel during the polymerization process. Subsequently, the gel is transformed into a solid structure via a freeze-drying process, which removes the liquid component while maintaining the solid structure’s integrity. If the water content is not frozen or if evaporation occurs before sublimation during freeze-drying, the aerogel’s porous structure may shrink, affecting its mechanical and thermal properties. After the process, the hydrogel samples were extracted gently from the moulds and frozen again in liquid nitrogen vapours until they reached a temperature of −70 °C. This instantly freezes the water content of the material, and negligible evaporation occurs, which could lead to the disintegration of the structure. This additional step is required because the freeze dryer maintains the low temperature in vacuum conditions but does not immediately reduce it from the sample. Rapid freezing is therefore carried out earlier. They are then placed in a freeze-dryer bottle at 0.002 mbar for 8 h to form the aerogel. The freeze-drying process sublimates the water ice crystals, leaving an empty spot (porous) without the collapse of the structure. Once ready, the samples were stored in closed envelopes in a dry environment to avoid contamination by humidity. If the water content is not frozen or if evaporation occurs before sublimation during freeze drying, the aerogel’s porous structure may shrink, affecting its mechanical and thermal properties. Overall, the aerogels produced seem identical at touch and sight, but they differ in weight and bulk density, which increase with the addition of LHS percentage (Section 3.3). The homogeneity distribution of LHS was confirmed throughout the SEM images of both the edge and core sections of the sample, validating the described procedure.

## 3. Characterization

The porous morphology was observed by using scanning electron microscopy (SEM) and via image processing techniques. The chemical analysis was performed by using Fourier transform infrared spectroscopy (FT-IR, Jasco FTIR-6600), and the test wavenumber range was 4000~400 cm^−1^. The X-ray diffractometer (XRD) was used to determine the crystal structure and phase composition of the aerogel by analysing the diffraction patterns produced by X-rays passing through the sample. The material properties were calculated in experimental ways following the literature on this topic, and eventually the thermal properties were measured with Hot Disk TPS 2500 S.

### 3.1. Porous Characterization

The porous morphology observed by using SEM showed how the simulant lunar regolith is homogeneously embedded and dispersed in the structure. Samples were inspected at different locations, particularly at the borders. The images obtained showed no variations in brightness or structure, indicating a homogeneous distribution of LHS within the samples. The SA/gelatin base develops a 3D framework with a randomly interconnected microporous structure.

**Figure 2:**
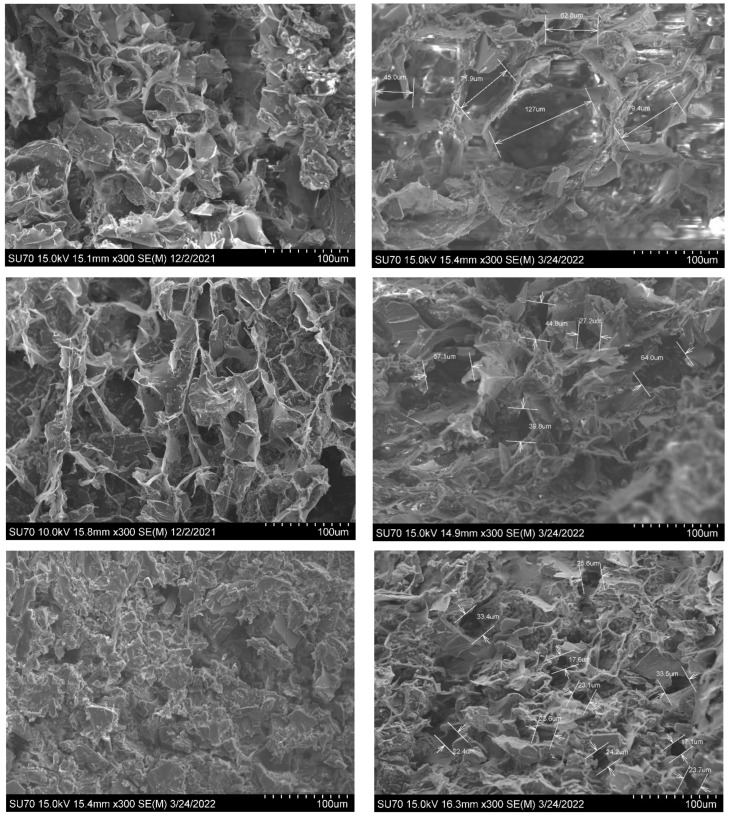
From the top: SEM ×300 images of aerogel with 20%/40%/60% of simulant lunar regolith from the highlands.

The production process preserved the skeleton of the starting hydrogels and minimised their shrinkage during drying. From these pictures, the minimum, mean and maximum diameter of porous material is reported and calculated in the table below (Table 3 and Table 4):

Overall, the simulant lunar regolith is homogeneously dispersed and distributed in all the samples. They present a sponge-like structure with porous dimensions on the order of micrometres. However, the pore dimensions considerably decrease at the increment of simulant lunar regolith, as shown in the figures above. The incorporation of LHS powder mass prevents the formation of larger porous, making the porous skeleton structure more robust. The structure presents an open-pore porosity that is gradually decreasing with increments in the percentage of simulant lunar regolith, as shown in Figure 2. This may be explained by the fact that the regolith particles insinuate into the empty spots, which are otherwise occupied by water, limiting the formation of larger pores. The phenomenon becomes even more evident for quantities greater than 40% (second row in Figure 2). The sponge-like structure appears more granular, and the lightweight property of aerogels is also compromised. Additionally, the pore distribution and the network structure become heterogeneous as the number of nanoparticles in the regolith increases.

### 3.2. Pore Characterization via Thresholding Methods of Image Processing

One of the most significant difficulties in determining the shape and size of porous materials is the time necessary for the procedure, combined with the possibility of restricted accuracy due to the analysis of only a small number of images. To address this challenge, an automated strategy that overcomes the limits of manual characterization techniques may be used. As a result, tools for automated image analysis, such as thresholding algorithms, have been developed to allow more efficient and accurate analysis of SEM pictures. A thresholding algorithm was implemented to analyse several SEM images of aerogels and automate the process. The segmentation [45] distinguishes the elements in the images into two parts: the pores and the solid matrix. The algorithm works by converting the SEM images, 10 per sample type in this case, into grayscale and then to a binary one, applying a threshold value to separate the pore areas from the solid matrix areas. The threshold value was determined based on the intensity values of the pixels in the image, and it was set to a value that provided the best separation between the pore and solid matrix areas. The algorithm automatically determined the threshold value based on the intensity values of the pixels in the image histogram. This provided the separation of the binary conversion 0 and 1 of the pixels below and above the threshold value, which translates into the best separation between the porous 1 and solid matrix area 0. Due to the nature of the grayscale original images, the open-source code [46] was not effective in porous or solid matrix detection. To this end, further morphological operations on the images to enhance the contrast and reverse their colours were implemented. In this way, 0 corresponds to porous material and 1 to the matrix, which helps the user’s visual inspection. The validation of the script was obtained by visually evaluating the pores detected through the watershed segmentation and their area approximation from the diameter of the SEM pictures. Eventually, their distribution will be represented in datasets.

**Figure 3:**
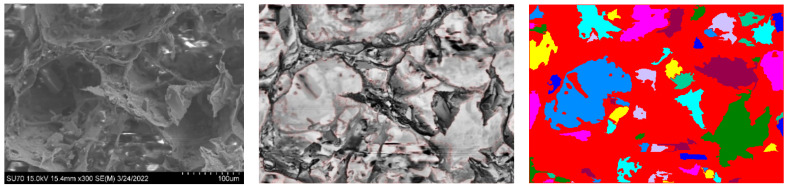
SEM (right) and processed (left) images with red markers defining the porous perimeters of an aerogel with 20% of simulant lunar regolith.

Figure 3 shows the steps: first, the initial SEM picture on the left; then, the use of markers to highlight different pore borders; and finally, the assignment of random colours to indicate each identified border from the processed image.

The conversion of the measurement of units is also integrated in the script, and it generates a table reporting the area and the diameter of each detected porous.

**Figure 4:**
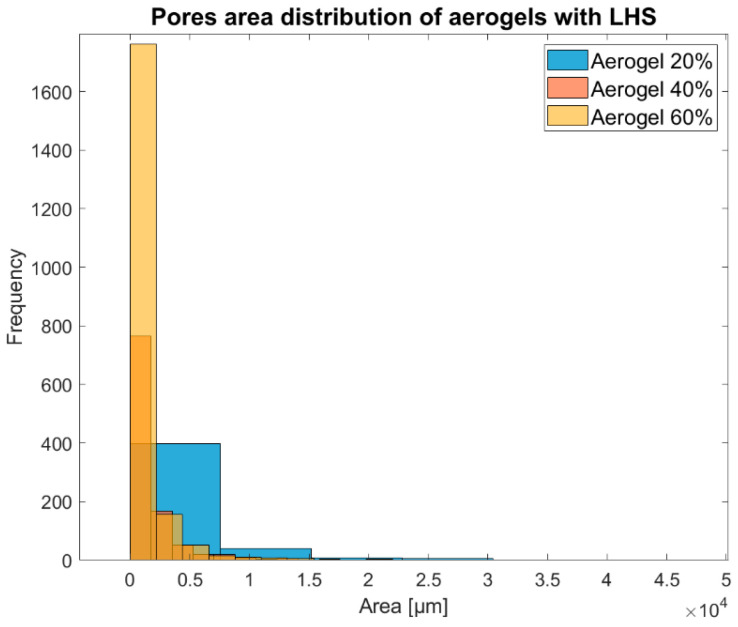
Distribution of porous material in the aerogel samples.

Despite the high degree of pore connectivity and the open porous interconnection, this approach provides a preliminary description of the pore identification and characteristics. Table 5 and Figure 4 show how a lower percentage of LHS in the samples will result in lower accuracy as the algorithm will primarily identify and prioritise smaller internal pores within the more obvious open-pore structure, overlooking larger pores.

Notwithstanding, it emphasises the importance of establishing the reliability of the automated detection process.

### 3.3. Thermal Properties

The thermal and material properties of the aerogels were calculated using Hot Disk 2500 S and reported in Table 6. All the thermal coefficients have a gradual increase related to the addition of simulant lunar regolith. While regolith is itself an insulator, the increase in thermal properties with higher LHS content is a result of the decrease in pore size. Pores are empty spaces within the material, and larger pore sizes lead to greater distances between opposite pore surfaces, resulting in enhanced thermal insulation. As the pores become smaller, the thermal insulation capability weakens, explaining the rise in the thermal properties’ values.

When it comes to concepts like Moon Village with its lunar structures to host facilities, lunar regolith is known to be a good thermal insulator and is the only resource available in situ. Although it reduces the thermal characteristics of SA/gelatine aerogels, it improves their mechanical properties, minimising deformation produced by intense heat sources in the harsh lunar environment.

The aerogels proposed in this paper are intended to contribute as innovative materials for Moon buildings, with an emphasis on enhancing insulating qualities. However, evaluating the most ideal material candidate for the harsh lunar environment becomes critical in the evaluation of material suitability. This involves a comprehensive trade-off analysis, taking into account various aspects, user needs, and application-specific requirements. It has been noted that on the ground, the inclusion of simulant lunar regolith improves mechanical stability to deformation caused by strong external heat loads, making it a significant component to consider for the overall performance and endurance of the buildings in such a hostile lunar environment.

### 3.4. Material Properties

The density of aerogels is a primary parameter to investigate because of the definition of the aerogel material itself. Table 7 presents the material parameters, which are explained in detail within this section. The dry mass and the bulk volume are the mass and the geometric volume, respectively, of the specimen after the freeze-drying process at room temperature. The estimation of the pore volume is obtained by subtracting the mass of the specimen soaked in water from the dry mass. Two terms used to characterise aerogels are bulk density and skeletal or apparent density [39,47]. The porosity, i.e., the ration of empty to total volume [48], and the following apparent density were calculated as per the method illustrated by Palacio et al. [49]. The apparent density was computed by dividing the dry mass by the difference between the geometric volume and the porous volume, whereas the bulk density represents the dry mass/geometric volume ratio.

### 3.5. Structural Characterisation

X-ray diffraction (XRD) and Fourier transform infrared (FTIR) spectroscopy are two common techniques used in materials science for structural characterization of materials.

#### 3.5.1. FTIR

**Figure 5:**
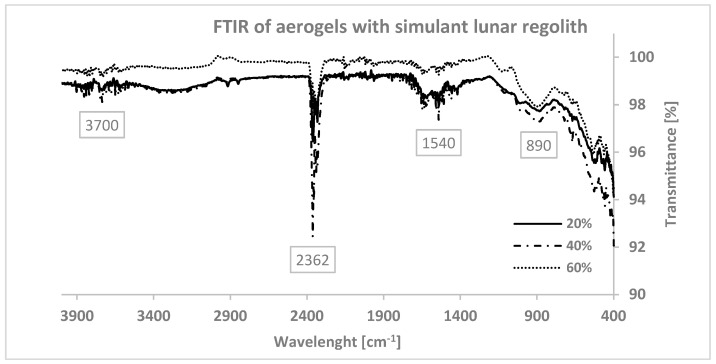
FTIR of aerogels with 20%, 40%, and 60% of LHS from the highlands.

To analyse the interactions between the components, changes to the chemical structure of SA, Gel, and simulant lunar regolith were investigated using FT/IR-6600 type A by Jasco, as shown in Figure 5. Spectrums were recorded using transmittance mode with a wavelength range of 4000–400 ${\mathrm{cm}}^{-1}$.

All the spectra show similar characteristics. There are five major areas, i.e., wavenumbers between 4000 and 3500 ${\mathrm{cm}}^{-1}$, 3500–3000 ${\mathrm{cm}}^{-1}$, 2300 ${\mathrm{cm}}^{-1}$, 1600–1400 ${\mathrm{cm}}^{-1}$, and 600–400 ${\mathrm{cm}}^{-1}$, which belong to the simulant lunar regolith compounds, SA and Gel. The spectral bands between 4000 and 3500 ${\mathrm{cm}}^{-1}$ are characteristic for hydroxyl groups (O–H; symmetrical stretching vibration) that are part of water molecules [50] and silanol groups (Si–OH), the latter mostly located at the surface of the silica particles [51]. In the gelatin spectrum, the broad peaks between 3500 and 3000 ${\mathrm{cm}}^{-1}$ are gradually attenuated as the percentage of simulant lunar regolith increases. They correspond to the Amide-A group [52], representing N-H stretching coupled with asymmetrical O–H stretching. The same tendency is also observed on peaks between 1600 and 1400 ${\mathrm{cm}}^{-1}$, caused by the bending vibrations of the alginate carboxylate group (-COOH). The sharpest peaks from the sample analysis are in the 2300 ${\mathrm{cm}}^{-1}$ range, whose elements appear in the simulant lunar regolith spectra, and they correspond to ${\mathrm{CO}}_{2}$ [53] absorbed by the aerogel. The presence of a strong C=C bending is confirmed in the 895–885 cm^−1^ range, as well as a strong halocarbon C-Cl stretching. The bands between 800 and 600 cm^−1^ are attributed to Al-O-Si vibrations [54]. In particular, between 534 and 541 ${\mathrm{cm}}^{-1}$ were related to Si–O asymmetrical bending vibrations, and 642–649 ${\mathrm{cm}}^{-1}$ were due to Al–O coordinate vibrations, indicating the occurrence of orthoclase feldspar [55]. This demonstrates that, despite an increase in the amount of regolith, the chemical bonds remain unchanged. However, the intensity of transmittance slightly decreases in the peaks due to the non-transparency of the samples. In other words, as the percentage of LHS increases in the samples, more IR light is absorbed, resulting in weaker peaks in the transmittance spectrum.

#### 3.5.2. XRD

XRD experiments were performed using the D8 Advance Eco by Bruker instrument with Cu (Kα) radiation operating at 40 kV and 25 mA in the range 5 to 70° 2θ, time per step 2 s, step 0.020°: 2θ. The software used to analyse the results is DIFFRAC SUIT, and the correlation of the peaks with the built-in library was matched by DIFFRAC.EVA V6.1.

XRD observations characterised a crystalline structure for all aerogels with a mild amorphism due to the gelatin and SA components [56] in the embedding hydrogel. The amorphism is more evident in samples with the lowest percentage of lunar regolith.

**Figure 6:**
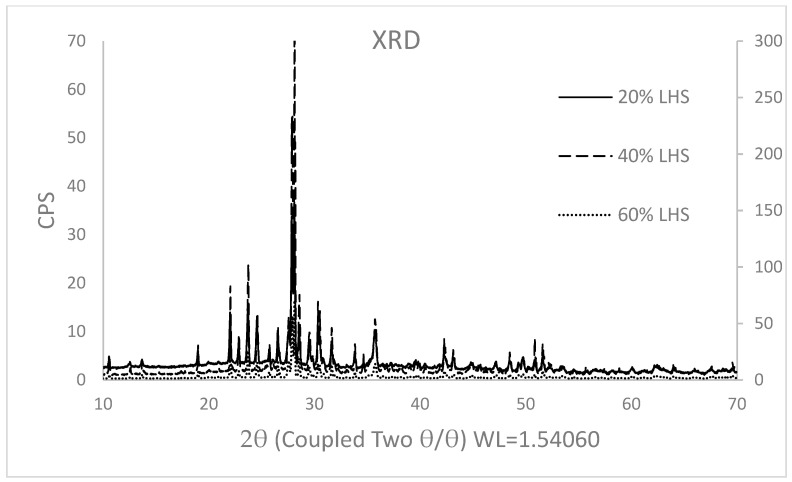
XRD graph of aerogels with 20%, 40%, and 60% of LHS. The 60% LHS data points have been plotted on the second axis, which is positioned to the left of the graph, with the specific purpose of enhancing clarity when differentiating the lines.

The highest peaks are registered between 2θ=22° and 2θ=36° (Figure 6) where the identification by similarity performed by the software DIFFRAC.EVA V6.1 shows their better match with the plagioclase triclinic family with a triclinic disposition, as confirmed in the LHS composition (Table 1) and the lunar soil characterization [57].

The width of the peaks is inversely related to the size of the crystal. A larger crystal equates to a thinner peak. The broader peak at the bottom of the graphs indicates that the sample is amorphous in nature, which is synonymous with a solid lacking perfect crystallinity [58], although its structure has been deeply impacted by the crystalline one of LHS. Hence, the percentages reported in Table 8.

The mechanical properties of crystals, which are groups of many particles (atoms, ions, or molecules) forming a regular lattice, depend on the forces of interaction between these particles. In each type of crystal—atomic, ionic, metallic, and molecular—the forces of particle interaction decrease with distance, with repulsive forces decreasing faster than attractive forces. The equilibrium distance between particles corresponds to the equality of attraction and repulsion forces. When the crystal is subjected to mechanical stresses, the balance of these forces is disturbed, the particles are displaced, and the lattice spacing changes. The resulting forces tend to bring the body back into equilibrium. The evidence supporting this observation is also reflected in the compression test results (Section 3.5.3). In the compression test, the initial linear part of the graph represents the material’s resistance to the stress load until the first microfracture occurs. Throughout the entire test, the material exhibits resistance to the external load, as evidenced by the fact that the complete fracture only occurs at the end of the graph. This behaviour indicates the material’s crystalline ability to withstand and endure external forces, showcasing its mechanical strength. Macroscopic changes in lattice spacing are manifested as elastic strains, and changes in particle interaction forces are manifested as stresses. This information can then be used to optimise properties of the aerogel for specific applications. As an example, amorphous structures can offer better mechanical flexibility compared to their crystalline counterparts. These results could be used as an advantage if the material is partially implemented in space structures. The lack of long-range molecular order allows the material to deform more easily [59], making it flexible and resilient when supporting loads, especially in a harsh environment. As polymer chains stretch and bend against each other, numerous attractive and repulsive forces influence how they arrange themselves, making them ordered [60]. This characteristic is beneficial in applications that require materials to withstand deformation and mechanical stress without failure.

The crystalline arrangement of atoms has been widely acknowledged to contribute to the mechanical properties of various components, including carbon. However, in the context of the aerogels studied here, it is important to view their amorphous behaviour not as a disadvantage but rather as an opportunity to tailor the material according to specific requirements and desired characteristics.

#### 3.5.3. Compression Test

Hardness tests were performed using Zwick Roell Z100 on square-shaped samples with a thickness between 4.12 and 7.41 mm. In a compression test, a compressive load is applied to a specimen. Deformation is measured as the load increases. The output data are force (*F*) and displacement (Δ*L*) values. The curves were later converted to stress (*σ*)−strain (*ϵ*) graphs according to Formulas (1) and (2). The specimen area is calculated from the produced specimen, and $L_{0}$ corresponds to the initial thickness of the specimen. Successively, the strain at fracture ($\epsilon_{f}$), and secant moduli (Young’s modulus or E-modulus) were determined using the equations:

(1) $\sigma =\frac{F}{A}$, for the stress value, defined by force (*F*) per unit area (*A*)
(2) $\epsilon =\frac{\Delta L}{L_{0}}$, for the strain value, given as a ration between the compression (ΔL) and the initial sample length ($L_{0}$)
(3) $E=\frac{\sigma }{\epsilon }$, for the Young’s modulus

**Figure 7:**
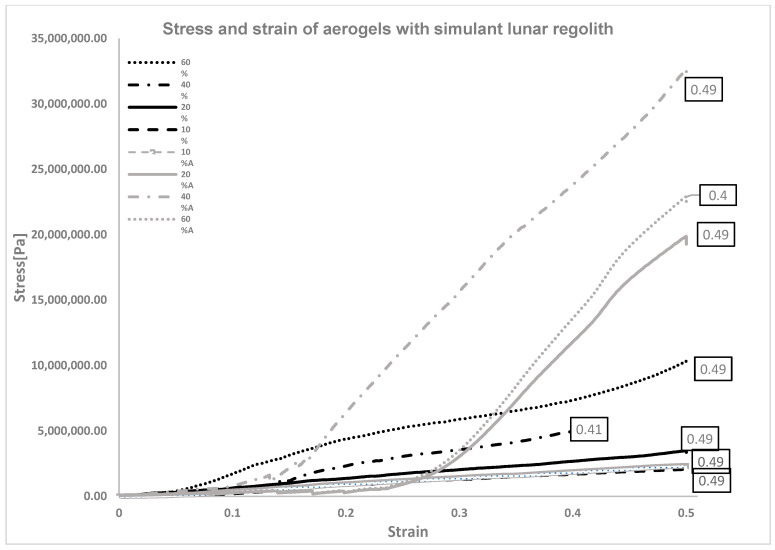
Comparative stress and strain curves of aerogels with 10%, 20%, 40%, and 60% of LHS after production and with ageing factor.

Overall, Figure 7 shows that the LHS acts as a reinforcement of the overall mechanical properties of the starting aerogel because of the linear increment of the Young’s modulus. The Young’s modulus measures a material’s stiffness and is directly proportional to its resistance to deformation under load. Therefore, the observed linear increment in the Young’s modulus indicates that the addition of LHS enhances the aerogel’s ability to withstand external forces and improves its mechanical strength. As the Young’s modulus increases, the material becomes stiffer and less prone to deformation, making it less fragile. As reported by S. P. Patil, P. Shendye, and B. Market [61], at least two regimes are evident: linear elastic and a yielding region. The E-modulus was calculated at strain values 0–0.04 (linear elastic) and 0.28–0.36 (yielding), where the specimens were less affected by the fractures and the stress and strain values had a linear tendency. For the aged samples, the regimes were considered between 0 and 0.12 (linear elastic) and 0.3–0.4 (yielding) values.

In the first column of the table, it is evident that there is no clear and linear increase in the Young’s modulus as the LHS percentage increases. This observation can be attributed to the presence of numerous microfractures in the material and the non-perfectly flat surface of the sample. However, in the second column (Table 9), a different trend emerges, with the Young’s modulus increasing by almost three times the initial value as the LHS content rises. This significant increment can be attributed to the abundant presence of LHS in samples with 60% LHS, which limits the formation of pores, as discussed in Section 3.1. Consequently, the smaller empty spots are less dominant, resulting in a more robust, solid structure that can better withstand external load stresses.

Due to the hydrophilic behaviour of the biopolymers SA and gelatine [62], the tests were carried out within three hours of extraction from the freeze-dryer bottles, which corresponds to the time taken to set up the machine and the running time of each test. The tests were also repeated after one week to investigate how humidity affected these characteristics while the specimens were stored in closed envelopes at room temperature. This parameter is significant since it relates to the material’s storage conditions, especially if it is produced on Earth.

The experimental results in Table 9 indicate that the aged samples exhibit almost 10 times more stiffness in the yielding regime due to the absorption of water molecules from the surrounding humidity. The water molecules penetrate easily into the hydrophilic matrix of the aerogels, and they create bonds with the hydroxyl group. Hydroxyl groups are polar groups consisting of an oxygen atom covalently bonded to a hydrogen atom. The bond between oxygen and hydrogen is highly polar due to the high electronegativity of the oxygen atom. As a result, the hydroxyl group strongly attracts water molecules, forming hydrogen bonds. In the linear elastic trend of the curve of the specimens, Figure 7 illustrates micro-fractures, which appear to be mitigated in the second set of experiments due to the higher absorption of humidity. Water molecules make the network structure more resistant to the compression load, increasing the E-modulus. In fact, the ageing factor critically reduces the yielding region and expands the elastic linear in the beginning, leaving the fracture point unvaried. This is caused by the fact that the sodium alginate (SA) molecular chain contains a large number of hydroxyl groups that strongly attract water molecules, forming hydrogen bonds. Therefore, alginate-based aerogels tend to absorb moisture easily, leading to the collapse of the aerogel structure [63]. Notably, SA gelatine aerogels stored in a non-dry environment became less rigid, indicating a potential increase in resistance to loads and hence the E-modulus. Therefore, proper storage of these materials in a dry environment is crucial to preserving their properties, as humidity absorption from the surroundings can impact the ageing factor and result in changes to the Young’s modulus. The tendency observed in aerogels with 10% simulant lunar regolith, reported in the graph only for reference reasons, validates the literature’s average E-modulus value of 1–2 MPa [64] for organic aerogels. It also demonstrates the increment of mechanical properties for samples with a higher percentage of LHS, whereas the strains at fracture remain unvaried for all the specimens. The two datasets examined show a similar pattern of stress-strain curves: freshly produced aerogels do not have an obvious yield point between the elastic and plastic parts, whereas those aged have, as evidenced by the sharp slope of the curves. Overall, for each dataset, the addition of LHS makes the specimen more resilient under load.

### 3.6. Electric Properties

Electrical properties such as resistance were measured with a DC vacuum tube multimeter hp, model 412A. Due to the high open porosity of the material, the resistance was observed to be greater than 5 GΩ.

## 4. Results

Generally, the properties of organic and inorganic aerogels vary, influenced by factors such as the materials used and the manufacturing methods implemented. Their suitability for space applications hinges on mission-specific needs and requirements, such as prioritising mechanical strength over insulation properties. The versatility of aerogels underscores their potential for addressing diverse challenges in space exploration. Hybrid aerogels, particularly those examined in this paper, have great potential for enhancing sustainability in space missions by utilising local resources as an insulator, such as regolith. These composite materials, which combine inorganic and organic components, allow for in situ resource utilisation, minimising the requirement for heavy insulating materials to be transported from Earth. Aerogels might potentially be manufactured on-site, suited to mission needs and local surroundings. This material can improve efficiency and contribute to better thermal management, lower energy consumption, and a longer lifespan for space equipment. The hybrid aerogels described in this paper may exhibit favourable insulation properties based on measurements of their insulation properties in Table 6 and the pore sizes estimated with SEM scanning and image analysis. However, additional modifications are necessary to ensure their suitability for use in space environments, such as hydrophobic treatments, if their production will happen on Earth. Furthermore, this characterization opens the opportunity for additional research under thermal vacuum conditions for calculating temperature gradients between the upper and lower surfaces. The LHS can be interpreted as a reinforcement of the mechanical properties of the starting hydrogel, making the final material less fragile; however in the need for an irreversible structure, the shape deformation under strong heat sources is yet to be mitigated. On the other hand, deformation is beneficial in applications where materials are required to withstand stress without failure. Depending on the user’s needs, the percentages evaluated show a tradeoff between the lightweight properties of the 20% LHS aerogels for the best insulation and the density properties of the less deformable at load 60% LHS aerogels. Overall, the mechanical and material properties increase proportionally as the LHS percentage increases, whereas the insulation properties decrease as the LHS percentage increases. The larger porous size of the sample with the lowest percentage of LHS limits the radiative part of the heat transfer through the material, benefiting the insulation capability. Among the three percentages of LHS, the 40% one represents a fair balance between the mechanical integrity and the material properties, such as density and porousness, without compromising the lightweight definition of aerogels. In the prospect of the new era of Moon exploration, the utilisation of in situ resources enables humans to make use of the resources available in space, cutting launch costs related to weight. Eventually, this research marked a contribution to the development, mechanical, and thermal analysis of future space structures on the Moon.

## Figures and Tables

**Table 1:** XRF Analysis of OPRH2N standard nearside highland regolith simulant [33] provided by Off Planet Research, LLC 2020 production company.

Chemical Components [% Mass]			
	Apollo 16 Highland Samples	OPRH2N	% Difference
${\mathrm{SiO}}_{2}$	45.10	47.89	2.79
${\mathrm{TiO}}_{2}$	0.60	0.52	−0.08
${\mathrm{Al}}_{2}O_{3}$	26.80	27.06	0.26
FeO	5.40	3.68	−1.72
MnO	0.22	0.06	−0.16
MgO	5.70	2.84	−2.86
CaO	15.60	14.19	−1.41
${\mathrm{Na}}_{2}O$	0.43	2.43	2.00
$K_{2}O$	0.14	0.25	0.11
$P_{2}O_{5}$	0.10	0.20	0.10
Sum	100.09	99.12	
LOI%		0.88	

**Table 2:** XRD composition of OPRH2N LHS provided by Off Planet Research.

Mineral Composition (Approximate)	Percentage [%]
Plagioclase	78
Amorphous Glass	14
Clinopyroxene	4
Olivine	3
Quartz	1 *
Ilmenite	Trace

* Anorthosite contains small amounts of crystalline silica (quartz) of 1% ± 1%.

**Table 3:** Mean porous diameter for respective samples.

Aerogel ID	Porous Medium Size [μm]
Aerogel 20%	77.22
Aerogel 40%	44.58
Aerogel 60%	24.42

**Table 4:** Pore diameter variation per sampling.

Sample ID	Minimum Porous Diameter [µm]	Maximum Porous Diameter [µm]
Aerogels 20% LSH	45	220
Aerogels 40% LSH	24.1	194
Aerogels 60% LSH	13.9	51.4

**Table 5:** Porous medium size diameter using watershed segmentation.

Aerogel ID	Porous Medium Size [μm]
Aerogel 20%	57.56
Aerogel 40%	35.94
Aerogel 60%	32.74

**Table 6:** Thermal properties of aerogels with increment of simulant lunar regolith.

ID Material	Thermal Conductivity [W/mK]	$\mathrm{Thermal}\;\mathrm{Diffusivity}\;[mm^{2}$/s]	Specific Heat [MJ/m^3^K]
Aerogel—0% LHS	0.063	0.25	0.25
Aerogel—20% LHS	0.07	0.22	0.34
Aerogel—40% LHS	0.11	0.29	0.39
Aerogel—60% LHS	0.13	0.28	0.44

Measures are a mean value out of 5 experimental measures on the same samples.

**Table 7:** Density analysis of aerogels with simulant lunar regolith.

ID Material	Dry Mass [g]	Bulk Volume [$cm^{3}$]	Poros Volume [$cm^{3}$]	Bulk Density [$g/cm^{3}$]	Apparent Density [$g/cm^{3}$]
Aerogel 20%	0.33	0.98	0.66	0.34	1.02
Aerogel 40%	0.34	0.78	0.51	0.58	1.56
Aerogel 60%	0.43	0.98	0.44	0.73	2.9

Simulant lunar regolith density measured through a compressed disk: 1.79 $g/{\mathrm{cm}}^{3}$.

**Table 8:** Amorphous behaviour of the aerogel samples with simulant lunar regolith from the highlands.

ID Material	Amorphous Behaviour [%]
Aerogel 20%	32.1
Aerogel 40%	18
Aerogel 60%	17.2

**Table 9:** Mechanical properties of aerogels with LHS.

ID Material	E-Modulus [MPa]—Linear Elastic (0–0.04)	E-Modulus [MPa]—Yielding (0.28–0.36)	E-Modulus [MPa]-Ageing—Linear Elastic (0–0.12)	E-Modulus [MPa]—Ageing—Yielding (0.3–0.4)	$\mathrm{Strain}\;\mathrm{at}\;\mathrm{Fracture},\;\epsilon_{f}$
Aerogel 10%	1.23	4.23	3.91	4.38	0.5
Aerogel 20%	5.59	5.84	2.42	90.04	0.49
Aerogel 40%	3.26	12.07	8.52	80.5	0.41
Aerogel 60%	4.86	13.37	2.79	103.84	0.46

## Data Availability

Not applicable.
